# The m6A modification of SOX18 leads to increased PTX3 and cardiomyocyte pyroptosis in sepsis-induced cardiomyopathy

**DOI:** 10.7150/thno.103809

**Published:** 2025-02-24

**Authors:** He Sun, Xinan Qiao, Xiangyan Peng, Hanzhao Zhu, Liyun Zhang, Liqing Jiang, Longteng Wang, Chao Xue, Jian Yang, Wei Yi, Bin Zhang, Jincheng Liu, Weixun Duan

**Affiliations:** 1 Department of Cardiovascular Surgery, The First Affiliated Hospital, The Air Force Medical University, 127 Changle West Road, Xi'an, Shaanxi 710032, China.; 2 School of Medicine, Northwest University, Xi'an, Shaanxi 710069, China.; 3 Department of Surgery, The 954th Hospital of the Chinese People's Liberation Army, Shannan, Tibet 856100, China.

**Keywords:** sepsis-induced cardiomyopathy, pyroptosis, PTX3, SOX18, N6-methyladenosine, RBM15, YTHDF2

## Abstract

**Rationale:** Sepsis-induced cardiomyopathy (SIC) is a rapidly progressing condition with poor prognosis in the absence of effective therapeutic interventions. Cardiomyocyte pyroptosis is a critical factor contributing to cardiac dysfunction in SIC. Currently, research on this mechanism remains unclear.

**Methods:** We performed LPS-induced primary mouse cardiomyocyte modeling and mouse SIC modeling. Through mRNA-Seq, we found significant pyroptosis in the cardiac tissue of SIC mice. Further confocal microscopy and immunoprecipitation results confirmed that PTX3 is an important participant in cardiomyocyte pyroptosis. We then used ChIP and dual-luciferase reporter assays to confirm that SOX18 exerts a transcriptional repression effect on PTX3. M6A-Seq and RNA stability assays confirmed that the m6A modification mediated/recognized by RBM15/YTHDF2 is a crucial factor in the changes of SOX18 in SIC.

**Results:** Our experiments demonstrated that the abnormally elevated PTX3 in SIC plays a key role in mediating pyroptosis. Under physiological conditions, PTX3 transcription is repressed by SOX18. However, during septic cardiomyopathy, SOX18 stability is compromised by RBM15/YTHDF2-mediated m6A modification, leading to increased PTX3 levels and the subsequent induction of cardiomyocyte pyroptosis.

**Conclusion:** In summary, we have delineated the RBM15/YTHDF2-SOX18-PTX3 axis in SIC. It provides a new approach for the treatment of cardiomyocyte pyroptosis in SIC and for improving prognosis.

## Introduction

Sepsis is a systemic inflammatory response triggered by infection, which, in severe cases, can lead to organ dysfunction and multiple organ failure^1^. Sepsis-induced cardiomyopathy (SIC) refers to cardiac dysfunction caused by sepsis. This condition is a life-threatening emergency that often progresses rapidly within a short period of time^2^. The inflammatory response induced by sepsis results in the release of a large number of inflammatory mediators, such as chemokines and interleukins, which damage myocardial cells and impair cardiac function. Additionally, hemodynamic disturbances further disrupt the normal function of other organs, severely compromising the patient's quality of life^3^. However, the pathogenesis of SIC is not yet fully understood, which poses a significant challenge to the development of effective therapies targeting SIC.

Pyroptosis is one of the cell death mechanisms most closely associated with SIC^4^. It is a type of inflammatory cell death characterized primarily by the loss of cell membrane integrity and the release of large amounts of inflammatory mediators. Pyroptosis is typically mediated by inflammasomes, such as the NLRP3 inflammasome, which activate Caspase-1, leading to the cleavage of Gasdermin D (GSDMD). This cleavage forms membrane pores, resulting in cell lysis and death, and the release of various inflammatory factors, including IL-1β and IL-18^5^. Existing studies have reported that inhibiting pyroptosis can effectively exert cardioprotective effects^6^.

PTX3 (Pentaxin 3), also known as long pentraxin 3, is an acute-phase protein that plays a significant role in immune regulation and inflammatory responses^7^. Additionally, studies have shown that PTX3 is closely associated with the occurrence and prognosis of cardiovascular events, such as myocardial infarction and heart failure^8^. Although the relationship between PTX3 and pyroptosis has been partially elucidated, the specific mechanisms underlying their interaction remain unknown^9^.

The SOX family (SRY-related HMG-box) is a group of transcription factors named for the presence of a highly conserved high mobility group (HMG) DNA-binding domain in its members^10^. SOX18, a member of the SOX family and part of the SOX F subfamily, is crucial for proper cardiac development, and its deficiency is often associated with cardiac malformations and the onset of various cardiovascular diseases^11^.

M6A (N6-methyladenosine) is a chemical modification widely present in eukaryotic mRNA and is one of the most abundant mRNA modifications identified to date. It plays a crucial role in various aspects of RNA metabolism. The dynamic regulation of m6A modification is coordinated by methyltransferases ("Writers"), demethylases ("Erasers"), and m6A-binding proteins ("Readers")^12^. Abnormal m6A modifications are associated with the progression and prognosis of SIC^13^.

In this study, we confirmed through both in vivo and cellular experiments that the key m6A enzymes RBM15 and YTHDF2 are abnormally elevated in SIC. Together, they drive the m6A modification of SOX18 mRNA, resulting in reduced mRNA stability and subsequent downregulation of SOX18 expression. The reduced levels of SOX18 fail to effectively suppress PTX3 transcription, leading to abnormally elevated PTX3 levels. This elevated PTX3 contributes to inflammasome formation and triggers pyroptosis in cardiomyocytes, ultimately resulting in impaired cardiac function.

## Results

### Elevated PTX3 in SIC contributes to the formation of the NLRP3 inflammasome

**Figure 1:**
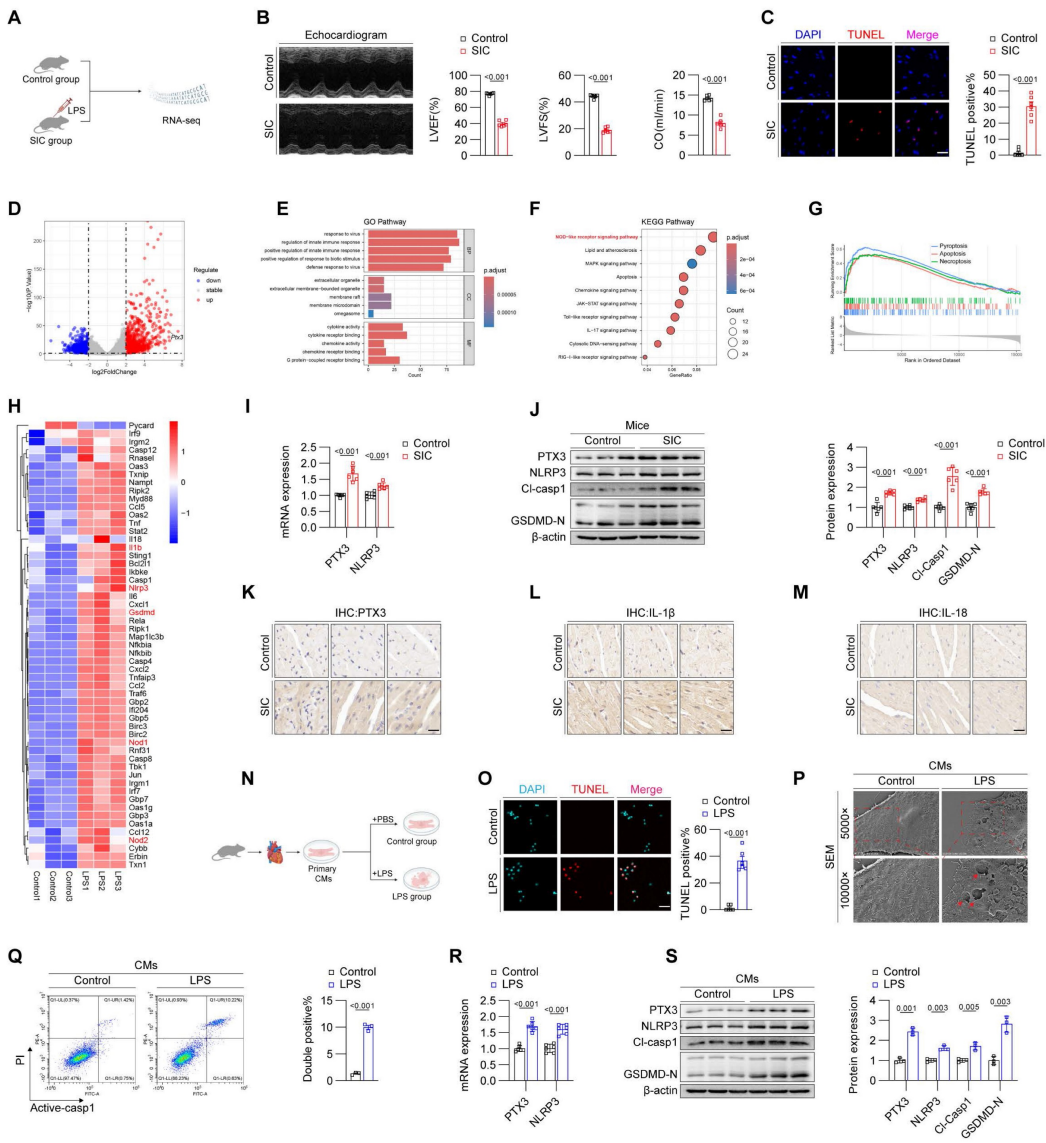
**Elevated PTX3 in SIC contributes to the formation of the NLRP3 inflammasome.** (A) Modeling strategy for Control and SIC mice groups. (B) Echocardiography results of mice (n = 6). (C) TUNEL staining results of cardiac tissue (n = 6). Scale bar: 25 μm. (D) Volcano plot displaying differentially expressed genes (n = 3). (E) GO enrichment analysis results. (F) KEGG pathway enrichment analysis results. (G) GSEA analysis results. (H) Heatmap showing pyroptosis-related genes. (I) qPCR detection of PTX3 and NLRP3 mRNA expression in cardiac tissue (n = 6). (J) Immunoblotting detection of PTX3, NLRP3, Cl-Casp1, and GSDMD protein expression in cardiac tissue (n = 6). (K, L, and M) Immunohistochemical detection of PTX3, IL-1β, and IL-18 expression in cardiac tissue. Scale bar: 20 μm. (N) Modeling strategy for primary cardiomyocytes from Control and LPS groups. (O) TUNEL staining results of primary cardiomyocytes (n = 6). Scale bar: 25 μm. (P) Scanning electron microscopy (SEM) results of primary cardiomyocytes. The red arrows indicate the membrane rupture observed during pyroptosis. (Q) Flow cytometry results for Active-Casp1 in primary cardiomyocytes (n = 3). (R) qPCR detection of PTX3 and NLRP3 mRNA expression in primary cardiomyocytes (n = 6). (S) Immunoblotting detection of PTX3, NLRP3, Cl-Casp1, and GSDMD protein expression in primary cardiomyocytes (n = 3).

To identify potential therapeutic targets for SIC, we divided C57BL/6J mice into a control group and an SIC group. The SIC group received LPS injections to induce SIC, while the control group received an equivalent volume of PBS (**Figure 1A**). After model establishment, we performed echocardiography to assess changes in cardiac function. The results demonstrated that, compared to the control group, mice in the SIC group exhibited a significant reduction in left ventricular ejection fraction (LVEF), fractional shortening (FS), and cardiac output (CO) (**Figure 1B**), indicating impaired cardiac systolic function in the SIC group. Additionally, the E/E' ratio in SIC mice increased, suggesting compromised diastolic function (**Figure S1A**). These findings confirm the effectiveness of the model and the significant cardiac dysfunction in the SIC group. Moreover, serum levels of myocardial injury markers CK-MB, cTnT, and LDH were markedly elevated in SIC mice (**Figures S1B, S1C, S1D**). Subsequently, we used the TUNEL assay to detect the total number of dead cells in the cardiac tissue of both groups. Fluorescence imaging revealed a significant increase in cell death in the SIC group compared to the control group, which showed nearly zero TUNEL-positive cells (**Figure 1C**). We then performed RNA-seq analysis on cardiac tissue from both groups. The sequencing results identified 785 upregulated and 715 downregulated genes, with PTX3 showing a marked change among all genes (**Figure 1D**). Gene Ontology (GO) analysis of these differentially expressed genes indicated a significant activation of immune responses (**Figure 1E**). KEGG pathway analysis highlighted the NOD-like receptor (NLR) signaling pathway, which is closely associated with pyroptosis (**Figure 1F**), and GSEA analysis further confirmed the abnormal activation of the pyroptosis in the myocardium of SIC mice, which exceeded the levels of apoptosis and necrosis, two common forms of cell death in SIC (**Figure 1G**). A heatmap illustrated the markedly increased expression of numerous pyroptosis-related core genes in the cardiac tissue of SIC mice (**Figure 1H**). Next, we used qPCR to measure the mRNA levels of PTX3 and NLRP3 in the cardiac tissue of both groups, confirming significantly elevated levels of both PTX3 and NLRP3 mRNA in the SIC group (**Figure 1I**). Western blot analysis showed increased PTX3 levels, along with elevated levels of NLRP3, cleaved Caspase-1 (Cl-Casp1), and GSDMD-N, which are key proteins involved in pyroptosis (**Figure 1J**). Immunohistochemistry further demonstrated the increased abundance of PTX3 and the inflammatory cytokines IL-1β and IL-18 in the cardiac tissue of SIC mice (**Figures 1K, 1L, and 1M**). Additionally, the levels of IL-1β and IL-18 in the serum of SIC mice were significantly elevated (**Figures S1E, S1F**).

Next, we extracted and treated primary cardiomyocytes from mice. Corresponding to the in vivo experiments, we divided the cardiomyocytes into a control group and an LPS-treated group (**Figure 1N**). The TUNEL assay confirmed that LPS treatment induced cell death in cardiomyocytes (**Figure 1O**). To identify the specific form of cell death, we used scanning electron microscopy to observe the cardiomyocytes in both groups. Under the electron microscope, the control group's cardiomyocytes displayed dense and intact cell membranes, while the LPS-treated group's cardiomyocytes exhibited pyroptosis-characteristic membrane pores and content leakage (indicated by red arrows) (**Figure 1P**). Based on these morphological observations, we employed flow cytometry with dual staining for Active-Caspase-1 and PI, which confirmed the occurrence of cell death in the LPS-treated group (**Figure 1Q**). Subsequent qPCR and Western blot analyses further validated the elevation of PTX3 and core pyroptosis molecules in the LPS-treated cardiomyocytes (**Figures 1R and 1S**), consistent with the in vivo results (**Figures 1I and 1J**).

Given the ongoing identification of proteins involved in inflammasome complex formation, along with the observed positive correlation between PTX3, pyroptosis markers, and the rate of pyroptosis in both in vivo and in vitro experiments, we hypothesized that PTX3 might be a component of the NLRP3 inflammasome. We used confocal laser scanning microscopy to examine the expression and localization of NLRP3, PTX3 and ASC in cardiomyocytes from both the control and LPS-treated groups. The results showed a significant increase in these proteins in the LPS-treated group compared to the control group. Notably, co-localization of these proteins was observed (indicated by white arrows) (**Figure S1G**). This hypothesis was further validated by co-immunoprecipitation (Co-IP) experiments (**Figure S1H**). The above results confirm that PTX3 is involved in the formation of the NLRP3 inflammasome.

### Inhibition of PTX3 effectively attenuates pyroptosis in cardiomyocytes during SIC

**Figure 2:**
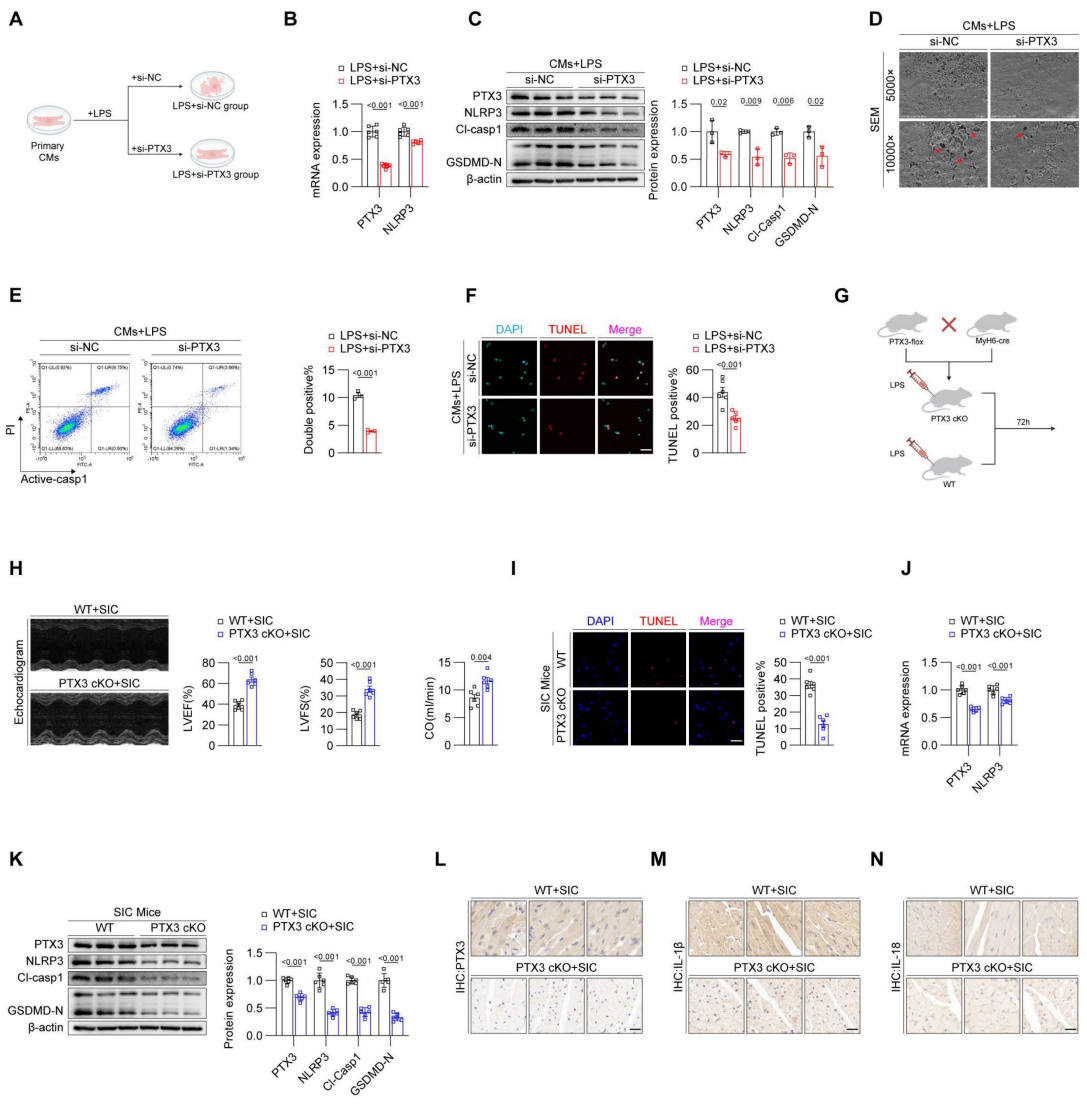
** Inhibition of PTX3 effectively attenuates pyroptosis in cardiomyocytes during SIC.** (A) Modeling strategy for LPS+si-NC and LPS+si-PTX3 groups of primary cardiomyocytes. (B) qPCR detection of PTX3 and NLRP3 mRNA expression in primary cardiomyocytes (n = 6). (C) Immunoblotting detection of PTX3, NLRP3, Cl-Casp1, and GSDMD protein expression in primary cardiomyocytes (n = 3). (D) SEM results of primary cardiomyocytes. The red arrows indicate the membrane rupture observed during pyroptosis. (E) Flow cytometry results for Active-Casp1 in primary cardiomyocytes (n = 3). (F) TUNEL staining results of primary cardiomyocytes (n = 6). Scale bar: 25 μm. (G) Modeling strategy for PTX3 cKO+SIC and WT+SIC groups of mice. (H) Echocardiography results of mice (n = 6). (I) TUNEL staining results of cardiac tissue (n = 6). Scale bar: 25 μm. (J) qPCR detection of PTX3 and NLRP3 mRNA expression in cardiac tissue (n = 6). (K) Immunoblotting detection of PTX3, NLRP3, Cl-Casp1, and GSDMD-N protein expression in cardiac tissue (n = 6). (L, M, and N) Immunohistochemical detection of PTX3, IL-1β, and IL-18 expression in cardiac tissue. Scale bar: 20 μm.

To confirm the impact of PTX3 on cardiomyocyte pyroptosis, we divided primary cardiomyocytes from mice into two groups: LPS+si-NC and LPS+si-PTX3 (**Figure 2A**). qPCR results showed that, compared to the LPS+si-NC group, the mRNA levels of PTX3 and NLRP3 were reduced in the LPS+si-PTX3 group (**Figure 2B**). Western blot analysis confirmed successful knockdown of PTX3 at the protein level, along with a decrease in key pyroptosis-related proteins (**Figure 2C**), the laser confocal results show that interfering with PTX3 effectively inhibits the formation of NLRP3 inflammasomes (**Figure S2A**). This further confirms that PTX3 not only participates in the formation of the inflammasome but also induces the upregulation of NLRP3 and the occurrence of pyroptosis. Scanning electron microscopy revealed that the LPS+si-PTX3 group had more intact cell membranes compared to the LPS+si-NC group, indicating less pyroptosis (**Figure 2D**). Flow cytometry and TUNEL staining further confirmed that PTX3 knockdown effectively reduced LPS-induced cell death in cardiomyocytes (**Figures 2E and 2F**). These results demonstrate that inhibiting PTX3 can significantly suppress LPS-induced pyroptosis in cardiomyocytes.

To investigate whether knocking down PTX3 in vivo can inhibit pyroptosis in cardiomyocytes, we crossed PTX3-flox mice with MyH6-cre mice to generate PTX3 Flox, MyH6-Cre (PTX3 cKO) homozygous mice, specifically knocking out PTX3 in cardiomyocytes of mice. Wild-type (WT+SIC) mice were used as the control group (**Figure 2G**). Echocardiography results indicated that, compared to the WT+SIC group, the cardiac function of the PTX3 cKO+SIC group was improved (**Figure 2H, Figure S2B**). Additionally, the TUNEL staining positive rate in myocardial tissue of the PTX3 cKO+SIC group was reduced compared to the WT+SIC group (**Figure 2I**). Both qPCR (Figure 2J) and western blot analysis (**Figure 2K**) confirmed that the knockdown of PTX3 effectively inhibited pyroptosis in the myocardial tissue of SIC mice, with subsequent immunohistochemistry results supporting the same conclusion (**Figure 2L-2N**). Serum levels of the myocardial injury markers CK-MB, cTNT, and LDH were significantly lower in the PTX3 cKO+SIC group compared to the WT+SIC group (**Figures S2C, S2D, S2E**). Additionally, although the mean serum levels of IL-1β and IL-18 were reduced in the PTX3 cKO+SIC group, the differences were not statistically significant (**Figures S2F, S2G**).

### Downregulation of SOX18 leads to increased PTX3 in SIC

**Figure 3:**
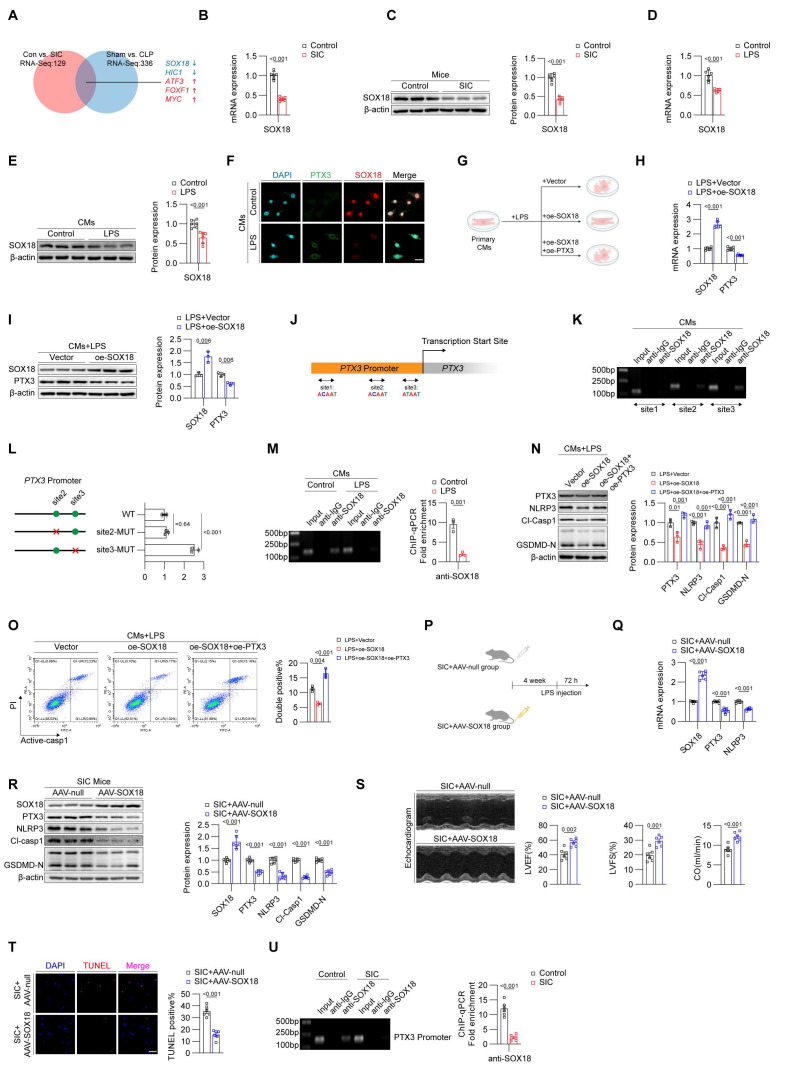
** Downregulation of SOX18 leads to increased PTX3 in SIC.** (A) Screening for transcription factors that may regulate PTX3. (B) qPCR analysis of SOX18 mRNA expression in mouse cardiac tissue (n = 6). (C) Western blot detection of SOX18 protein expression in mouse cardiac tissue (n = 3). (D) qPCR detection of SOX18 mRNA expression in primary mouse cardiomyocytes (n = 6). (E) Western blot detection of SOX18 protein expression in primary mouse cardiomyocytes (n = 3). (F) Immunofluorescence analysis of PTX3 and SOX18 expression in primary mouse cardiomyocytes. Scale bar: 25 μm. (G) Modeling strategy for LPS+Vector, LPS+oe-SOX18, and LPS+oe-SOX18+oe-PTX3 groups in primary cardiomyocytes. (H) qPCR analysis of SOX18 and PTX3 mRNA expression in primary mouse cardiomyocytes (n = 6). (I) Western blot detection of SOX18 and PTX3 protein expression in primary mouse cardiomyocytes (n = 3). (J) Schematic of PTX3 promoter and specific primer design. (K) ChIP-PCR results in primary mouse cardiomyocytes. (L) Plasmid construction schematic and dual-luciferase reporter assay (n = 3). (M) ChIP-qPCR results for site 3 in primary mouse cardiomyocytes (n = 3). (N) Western blot detection of PTX3, NLRP3, Cl-Casp1, and GSDMD-N protein expression in mouse cardiac tissue (n = 3). (O) Flow cytometry analysis of Active-Casp1 in primary mouse cardiomyocytes (n = 3). (P) Modeling strategy for SIC+AAV-null and SIC+AAV-SOX18 groups of mice. (Q) qPCR detection of SOX18, PTX3, and NLRP3 mRNA expression in mouse cardiac tissue (n = 6). (R) Western blot detection of SOX18, PTX3, NLRP3, Cl-Casp1, and GSDMD-N protein expression in mouse cardiac tissue (n = 6). (S) Echocardiography results of mice (n = 6). (T) TUNEL staining results of mouse cardiac tissue (n = 6). Scale bar: 25 μm. (U) ChIP-qPCR results for site 3 in mouse cardiac tissue (n = 3).

Given that RNA-seq and qPCR analyses have confirmed that the increase of PTX3 occurs primarily at the mRNA level, we focused on identifying potential transcriptional regulators of PTX3. Using RNA-Seq data from both CLP- and LPS-induced mouse SIC models, combined with databases such as JASPAR and CISBP, we identified five potential transcription factors, including SOX18, HIC1, ATF3, FOXF1, and MYC, that may regulate PTX3 expression (Figure 3A). Among them, SOX18 exhibited the most significant changes and has been reported to be associated with the prognosis of SIC^14^. We validated the reduction of SOX18 in the cardiac tissue of SIC mice using qPCR (**Figure 3B**) and Western blotting (**Figure 3C**), confirming a marked decrease in SOX18 expression in SIC mice. Similarly, qPCR and Western blotting of primary cardiomyocytes from control and LPS-treated mice also showed a decrease in SOX18 expression, consistent with in vivo findings (**Figures 3D and 3E**). Immunofluorescence results further confirmed the inverse relationship between SOX18 and PTX3 expression in LPS-treated cardiomyocytes (**Figure 3F**). To investigate the function of SOX18, we transfected primary cardiomyocytes treated with LPS using empty vectors and SOX18 overexpression plasmids (**Figure 3G**). qPCR (**Figure 3H**) and Western blotting (**Figure 3I**) revealed that overexpression of SOX18 resulted in reduced PTX3 expression, suggesting that SOX18 negatively regulates PTX3.

However, HIC1, ATF3, FOXF1, and MYC did not show regulatory effect on PTX3 (**Figure S3A**). We then obtained the PTX3 promoter sequence and designed specific primers around three different sites predicted by FOMO tool (**Figure 3J**). ChIP-PCR results confirmed that under physiological conditions, SOX18 binds near the transcription start site of the PTX3 promoter (site 3) (**Figure 3K**). Considering that site1 showed negative results in ChIP experiments, we constructed WT, site2-MUT, and site3-MUT plasmids, through dual-luciferase reporter assays, we identified that site3 on the PTX3 promoter is the key site where SOX18 exerts its transcriptional repression effect (**Figure 3L**). However, LPS treatment significantly weakened the binding of SOX18 to the PTX3 promoter (**Figure 3M**). Furthermore, we co-transfected cardiomyocytes stimulated with LPS using SOX18 overexpression and PTX3 overexpression plasmids. Western blot analysis showed that SOX18 overexpression significantly reduced the levels of pyroptosis-related proteins, while PTX3 overexpression reversed this effect (**Figure 3M**). Subsequent flow cytometry (**Figure 3O**) confirmed that SOX18 overexpression effectively inhibited cardiomyocyte death, but this protective effect was abolished upon PTX3 overexpression. After clarifying the regulatory relationship between SOX18 and PTX3, we considered potential transcriptional cofactors that might act synergistically with SOX18. MEF2C has been reported in the literature^15^, but we found that its expression did not show significant changes in LPS-treated cardiomyocytes (**Figure S3B**).

To investigate whether SOX18 exhibits a similar protective effect in vivo, we designed an adeno-associated virus 9 carrying the cTNT promoter and the SOX18 CDS region to specifically overexpress SOX18 in the cardiomyocytes of SIC model mice (SIC+AAV-SOX18 group), with the SIC+AAV-null group serving as a control (**Figure 3P**). qPCR and Western blot analyses confirmed that overexpression of SOX18 in vivo effectively reduced the levels of PTX3, NLRP3, GSDMD-N, and Cl-CASP1 in myocardial cells (**Figures 3Q and 3R**). Echocardiography results showed that SOX18 overexpression significantly alleviated the cardiac dysfunction induced by SIC (**Figure 3S, Figure S3A**). Additionally, TUNEL staining demonstrated that SOX18 overexpression markedly decreased the cell death rate in the cardiac tissue of SIC mice (**Figure 3T**). In the SIC+AAV-SOX18 group, serum levels of cardiac injury markers CK-MB, CTNT, and LDH were significantly reduced compared to the SIC+AAV-null group (**Figures S3B, S3C, S3D**). Moreover, the average levels of IL-1β and IL-18 in the SIC+AAV-SOX18 group were lower; however, these reductions were not statistically significant (**Figures S3E, S3F**). Subsequent ChIP assays confirmed that the binding of SOX18 to the PTX3 promoter was significantly reduced in the cardiac tissue of SIC mice (**Figure 3U**), consistent with in vitro results. These findings suggest that the transcription factor SOX18 exerts cardioprotective effects by binding to the PTX3 promoter region, thereby inhibiting PTX3 transcription and subsequent cardiomyocyte pyroptosis. This protective effect is notably diminished in SIC due to the downregulation of SOX18 expression.

### Downregulation of SOX18 is driven by RBM15-mediated m6A modification

**Figure 4:**
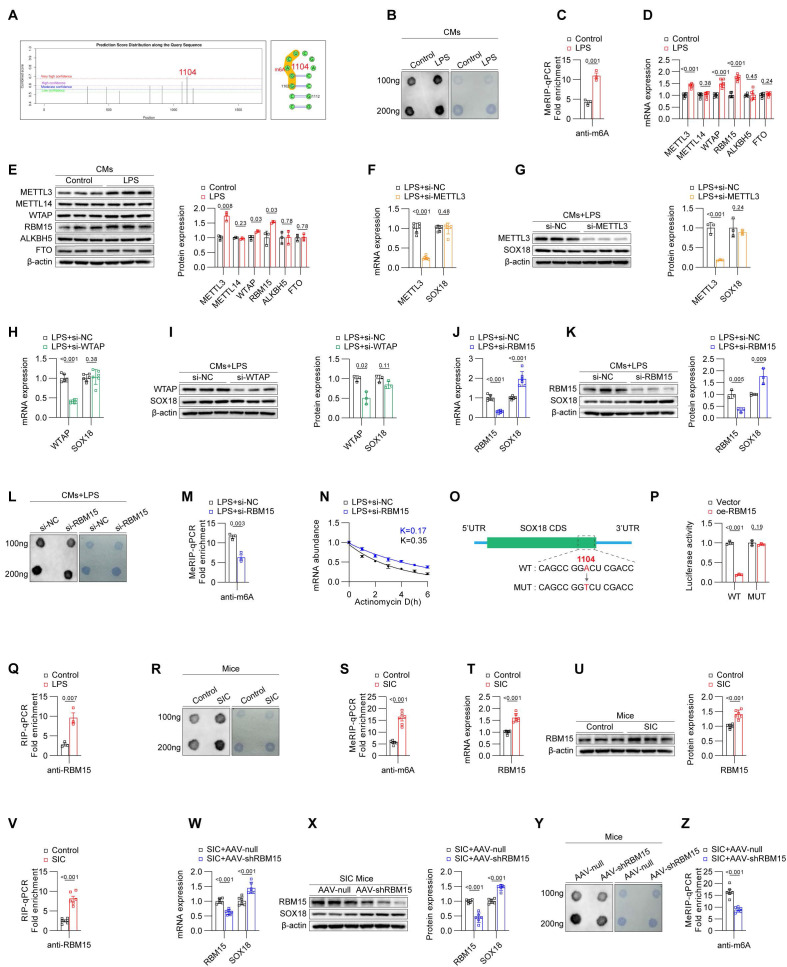
** Downregulation of SOX18 is driven by RBM15-mediated m6A modification.** (A) Prediction of m6A sites on SOX18 mRNA. (B) Dot blot analysis of global m6A modification levels in primary mouse cardiomyocytes. (C) MeRIP-qPCR detection of m6A modification levels on SOX18 mRNA in primary mouse cardiomyocytes (n = 3). (D) qPCR analysis of METTL3, METTL14, WTAP, and RBM15 mRNA expression in primary mouse cardiomyocytes (n = 6). (E) Western blot detection of METTL3, METTL14, WTAP, and RBM15 protein expression in primary mouse cardiomyocytes (n = 3). (F-K) Changes in SOX18 expression detected by qPCR and Western blot after knockdown of METTL3, METTL14, WTAP, and RBM15 in LPS-treated primary mouse cardiomyocytes. (L) Dot blot analysis of global m6A modification levels in primary mouse cardiomyocytes. (M) MeRIP analysis of SOX18 mRNA m6A modification levels in primary mouse cardiomyocytes (n = 3). (N) RNA stability experiment for SOX18. (O) Schematic of SOX18 mRNA m6A site mutations. (P) Dual-luciferase reporter assay. (Q) RIP-qPCR results of primary mouse cardiomyocytes (n = 3). (R) Dot blot analysis of global m6A modification levels in mouse cardiac tissue. (S) MeRIP-qPCR detection of SOX18 mRNA m6A modification levels in mouse cardiac tissue (n = 3). (T) qPCR analysis of RBM15 mRNA expression in primary mouse cardiomyocytes (n = 6). (U) Western blot detection of RBM15 protein expression in mouse cardiac tissue (n = 6). (V) RIP-qPCR results of mouse cardiac tissue (n = 3). (W) qPCR detection of RBM15 and SOX18 mRNA expression in mouse cardiac tissue (n = 6). (X) Western blot detection of RBM15 and SOX18 protein expression in mouse cardiac tissue (n = 6). (Y) Dot blot analysis of global m6A modification levels in mouse cardiac tissue. (Z) MeRIP-qPCR detection of SOX18 mRNA m6A modification levels in mouse cardiac tissue (n = 6).

N6-methyladenosine (m6A) modification is a widespread and functionally significant epigenetic mark. Our research group has previously identified m6A modifications in various cardiovascular diseases, such as atrial fibrillation and diabetic cardiomyopathy, and demonstrated that m6A modifications play a crucial role in the progression of these conditions. We hypothesized that m6A modification may similarly influence the SIC-SOX18-PTX3 pathway. To test this hypothesis, we analyzed the mRNA sequence of SOX18 using the SRAMP prediction tool, which revealed potential m6A modification sites on SOX18 mRNA, and the highest-scoring site is located at base A at position 1104 (**Figure 4A**). Next, we employed Dot Blot assays to compare the global m6A modification levels between control and LPS-treated primary cardiomyocytes from mice. The results indicated a significant increase in global m6A levels in the LPS group compared to the control group (**Figure 4B**). Moreover, MeRIP-qPCR analysis confirmed an elevated m6A modification level on SOX18 mRNA in the LPS-treated cardiomyocytes (**Figure 4C**). To identify the driver of the aberrantly elevated m6A levels in LPS-treated cardiomyocytes, we assessed the expression levels of key m6A "writers" and "erasers" in both control and LPS groups. qPCR and Western blot results revealed increased levels of METTL3, WTAP, and RBM15 in the LPS group (**Figures 4D and 4E**). We then performed siRNA-mediated knockdown of these "writers" in LPS-treated cardiomyocytes. The results demonstrated that knockdown of RBM15 significantly upregulated SOX18 expression, while knockdown of METTL3 and WTAP did not affect SOX18 expression (**Figures 4F-4K**). Subsequent Dot Blot and MeRIP-qPCR analyses confirmed that RBM15 knockdown effectively reduced the overall m6A modification levels (**Figure 4L**) and the m6A modification on SOX18 mRNA (**Figure 4M**) in LPS-treated cardiomyocytes, while interference with METTL3 or WTAP did not significantly affect the m6A modification levels of SOX18 mRNA (**Figure S4A**). Actinomycin D assays further demonstrated that RBM15 knockdown enhanced SOX18 mRNA stability, providing additional evidence that RBM15 influences SOX18 expression by reducing SOX18 mRNA stability (**Figure 4N**). To further pinpoint the specific m6A modification sites, we performed site-directed mutagenesis on SOX18 mRNA (**Figure 4O**) followed by a dual-luciferase reporter assay (**Figure 4P**). The fluorescence intensity, which reflects SOX18 mRNA stability, confirmed that mutating the m6A sites on SOX18 mRNA abolished the impact of RBM15 expression on SOX18 mRNA stability. Finally, RIP-qPCR analysis demonstrated that the binding of RBM15 to SOX18 mRNA was significantly enhanced in LPS-treated cardiomyocytes compared to the control group (**Figure 4Q**).

We then examined the levels of m6A modification in cardiac tissues from both the control and SIC groups. The results demonstrated that, compared to the control group, the SIC group exhibited a significant increase in overall m6A modification levels (**Figure 4R**) as well as in m6A modification on SOX18 mRNA (**Figure 4S**). Furthermore, both the mRNA (**Figure 4T**) and protein levels (**Figure 4U, Figure S4B**) of RBM15 were elevated, consistent with the in vitro findings. RIP-qPCR analysis confirmed that the binding of RBM15 to SOX18 mRNA was enhanced in the cardiac tissues of the SIC group (**Figure 4V**). When RBM15 expression was specifically knocked down in myocardial cells of SIC mice using AAV-CTNT-shRBM15, there was an observed increase in SOX18 expression (**Figures 4W and 4X**), accompanied by a decrease in overall m6A modification levels (**Figure 4Y**) and m6A modification on SOX18 mRNA (**Figure 4Z**).

### YTHDF2 influences the stability of SOX18 mRNA

**Figure 5:**
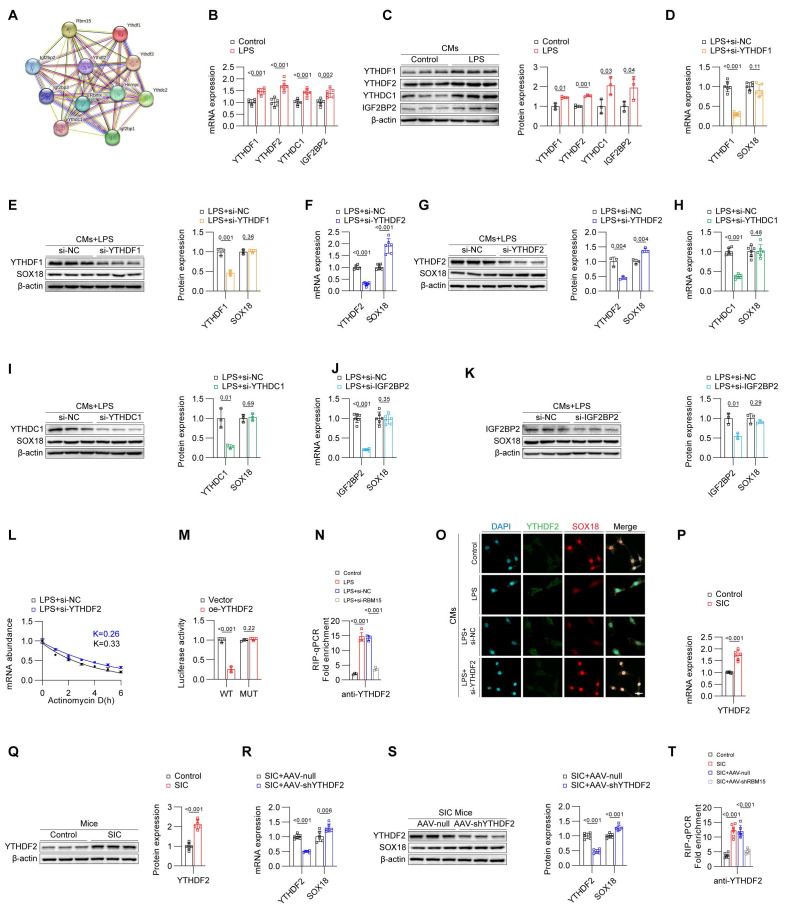
** YTHDF2 influences the stability of SOX18 mRNA.** (A) m6A readers associated with RBM15. (B) qPCR detection of YTHDF1, YTHDF2, YTHDC1, and IGF2BP2 mRNA expression in primary mouse cardiomyocytes (n = 6). (C) Western blot detection of YTHDF1, YTHDF2, YTHDC1, and IGF2BP2 protein expression in primary mouse cardiomyocytes (n = 3). (D-K) Changes in SOX18 expression detected by qPCR and Western blot after knockdown of YTHDF1, YTHDF2, YTHDC1, and IGF2BP2 in LPS-treated primary mouse cardiomyocytes. (L) RNA stability assay for SOX18. (M) Dual-luciferase reporter assay. (N) RIP-qPCR results of primary mouse cardiomyocytes (n = 3). (O) Immunofluorescence analysis of YTHDF2 and SOX18 expression in primary mouse cardiomyocytes. Scale bar: 25 μm. (P) qPCR detection of YTHDF2 mRNA expression in mouse cardiac tissue (n = 6). (Q) Western blot detection of YTHDF2 mRNA expression in mouse cardiac tissue (n = 6). (R) qPCR detection of YTHDF2 and SOX18 mRNA expression in mouse cardiac tissue (n = 6). (S) Western blot detection of YTHDF2 and SOX18 protein expression in mouse cardiac tissue (n = 6). (T) RIP-qPCR results of mouse cardiac tissue (n = 3).

In the biological functions of m6A modifications, both m6A writers and readers play crucial roles; m6A writers are responsible for adding the modifications, while m6A readers recognize these modifications and regulate corresponding biological processes. Using the STRING database, we identified several potential m6A readers that might cooperate with RBM15 (**Figure 5A**). qPCR and Western blot analyses revealed that YTHDF1, YTHDF2, YTHDC1, and IGF2BP2 were all upregulated in primary mouse cardiomyocytes stimulated by LPS (**Figures 5B and 5C**). To identify the m6A reader that potentially acts on SOX18, we transfected LPS-stimulated cardiomyocytes with siRNAs targeting these readers. The results from qPCR and Western blot showed that silencing YTHDF2 effectively increased the expression of SOX18, whereas silencing other readers had no impact on SOX18 levels (**Figures 5D-5K**). Actinomycin D assays further confirmed that knocking down YTHDF2 enhanced the stability of SOX18 mRNA, suggesting that YTHDF2 may inhibit SOX18 expression by reducing the stability of SOX18 mRNA (**Figure 5L**). Similarly, when the m6A sites on SOX18 mRNA were mutated, changes in YTHDF2 levels no longer affected SOX18 mRNA stability (**Figure 5M**). RIP-qPCR experiments confirmed that LPS treatment increased the binding of YTHDF2 to SOX18 mRNA. Importantly, when RBM15 was silenced, the binding of YTHDF2 to SOX18 mRNA decreased, further indicating that RBM15-mediated m6A modification of SOX18 mRNA is recognized by YTHDF2, which drives the reduction of SOX18 mRNA stability (**Figure 5N**). The following rescue experiments confirmed that SOX18 knockdown reversed the decrease in PTX3 induced by RBM15 or YTHDF2 knockdown, while SOX18 overexpression inhibited the increase in PTX3 caused by RBM15 or YTHDF2 overexpression (**Figures S5A and S5B**). Additionally, YTHDF2 overexpression suppressed the SOX18 increase induced by RBM15 knockdown, while YTHDF2 knockdown reversed the SOX18 decrease caused by RBM15 overexpression (**Figure S5C**), confirming the co-regulatory effect of RBM15 and YTHDF2 on SOX18 expression. Immunofluorescence results demonstrated that knocking down YTHDF2 significantly improved SOX18 expression levels (**Figure 5O**). Subsequently, we validated our findings in vivo. Similar to the in vitro results, the mRNA and protein levels of YTHDF2 were elevated in the cardiac tissues of the SIC group (**Figures 5P and 5Q, Figure S5D**). Knocking down YTHDF2 in mouse cardiomyocytes using AAV-CTNT-shYTHDF2 effectively increased SOX18 expression in cardiac tissues (**Figures 5R and 5S, Figure S5E**). RIP-qPCR further confirmed that the elevated binding of YTHDF2 to SOX18 mRNA in the cardiac tissues of SIC mice was mediated by RBM15 (**Figure 5T**). These findings collectively demonstrate that in SIC, RBM15 catalyzes the m6A modification of SOX18 mRNA, and YTHDF2 recognizes these modifications, leading to decreased stability of SOX18 mRNA and reduced SOX18 expression.

### Inhibition of RBM15/YTHDF2 effectively attenuates pyroptosis in cardiomyocytes during SIC

Building on the results above, we established that RBM15 and YTHDF2 are key drivers of the downregulation of SOX18 (**Figures 4-5**). The reduction in SOX18 leads to an increase in PTX3, which contributes to pyroptosis in cardiomyocytes during SIC (**Figures 1-3**). Therefore, we hypothesized that inhibiting RBM15 and YTHDF2 could mitigate PTX3 expression and alleviate pyroptosis and cardiac dysfunction by improving SOX18 levels.

**Figure 6:**
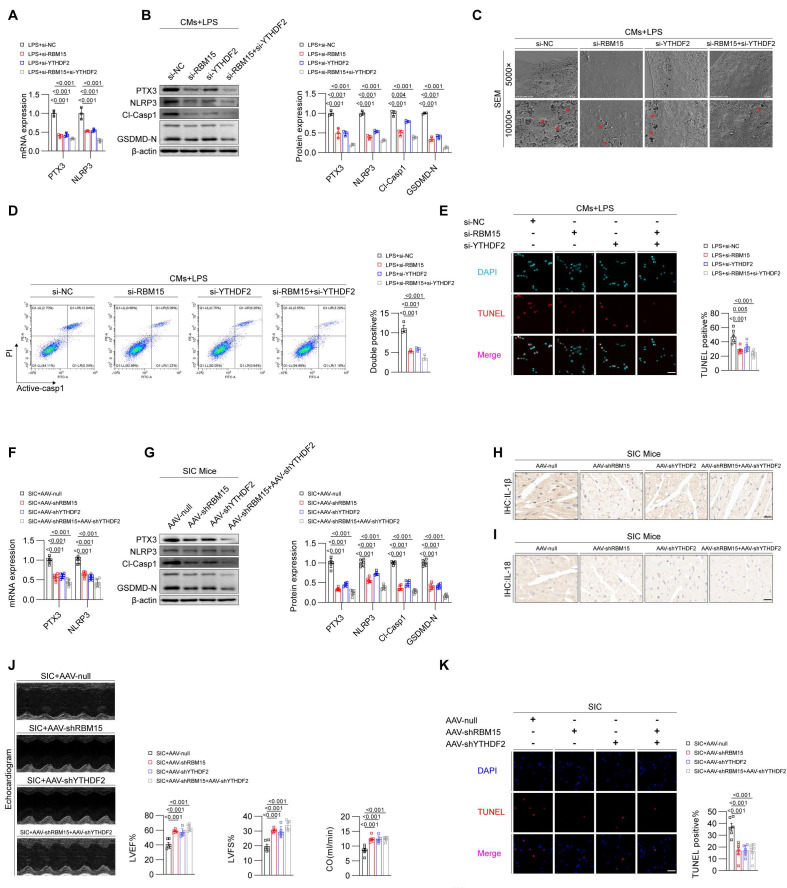
** Inhibition of RBM15/YTHDF2 effectively attenuates pyroptosis in cardiomyocytes during SIC.** (A) qPCR detection of PTX3 and NLRP3 mRNA expression in primary mouse cardiomyocytes (n = 6). (B) Western blot detection of PTX3 and NLRP3 protein expression in primary mouse cardiomyocytes (n = 3). (C) SEM results of primary mouse cardiomyocytes. The red arrows indicate the membrane rupture observed during pyroptosis. (D) Flow cytometry analysis of Active-Casp1 in primary mouse cardiomyocytes (n = 3). (E) TUNEL staining results of primary mouse cardiomyocytes (n = 6). (F) qPCR detection of PTX3 and NLRP3 mRNA expression in mouse cardiac tissue (n = 6). (G) Western blot detection of PTX3 and NLRP3 protein expression in mouse cardiac tissue (n = 3). (H, I) Immunohistochemistry analysis of IL-1β and IL-18 expression in mouse cardiac tissue. Scale bar: 20 μm. (J) Echocardiography results in mice (n = 6). (K) TUNEL staining results of mouse cardiac tissue (n = 6). Scale bar: 25 μm.

To test this hypothesis, we treated LPS-induced primary mouse cardiomyocytes with siRNAs targeting RBM15 and YTHDF2, either individually or in combination. qPCR and Western blot analyses showed that knocking down RBM15 and/or YTHDF2 significantly reduced the levels of PTX3 and pyroptosis markers (**Figures 6A and 6B**). Scanning electron microscopy revealed that RBM15 and YTHDF2 inhibition alleviated membrane perforation associated with pyroptosis (**Figure 6C**). Furthermore, flow cytometry (**Figure 6D**) and TUNEL staining (**Figure 6E**) confirmed that silencing RBM15 and YTHDF2 inhibited LPS-induced cell death in cardiomyocytes. We then assessed the effects of in vivo interference with RBM15/YTHDF2 on SIC mice. The results demonstrated that interference with RBM15/YTHDF2 effectively reduced the levels of pyroptosis markers in cardiac tissue of SIC mice (**Figures 6F-6I**), alleviated SIC-induced cardiac dysfunction (**Figure 6J, Figure S6A**), and attenuated myocardial injury (**Figure 6K, Figures S6B, S6C, S6D**). Additionally, it lowered the average levels of circulating pyroptosis markers (**Figures S6E, S6F**).

### Validation of the RBM15/YTHDF2-SOX18-PTX3 axis in the cecal ligation and puncture (CLP) model

**Figure 7:**
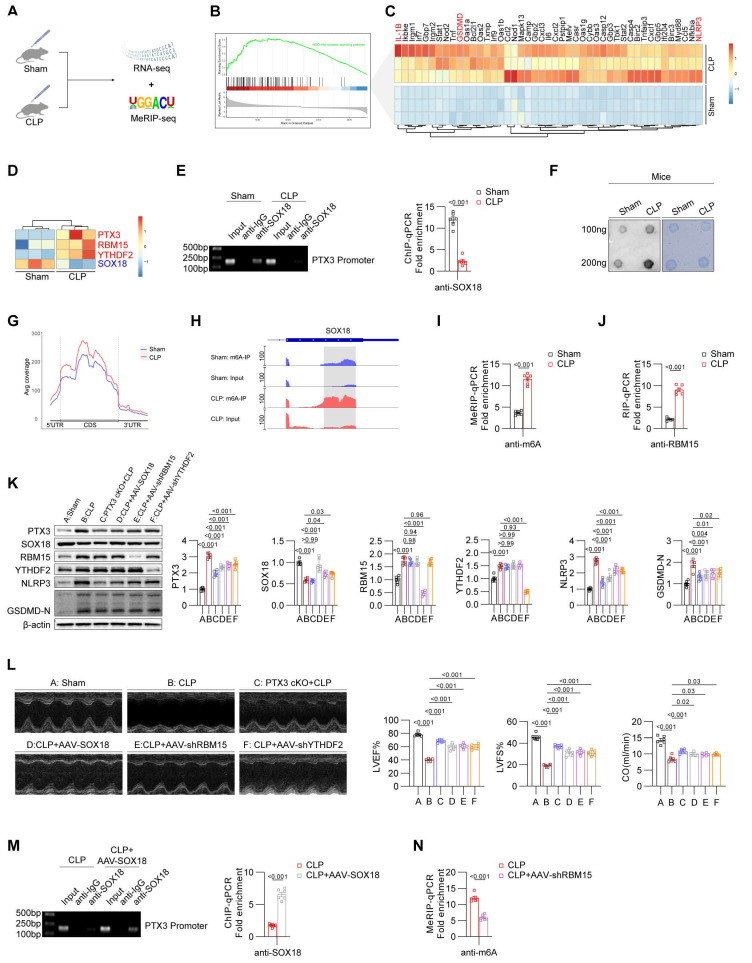
**Validation of the RBM15/YTHDF2-SOX18-PTX3 axis in the CLP model.** (A) Experimental design of Sham and CLP groups in mice. (B) GSEA analysis of pyroptosis-related pathway changes in the heart of CLP group mice. (C) Heatmap showing the differential expression of pyroptosis-related genes in the heart of Sham and CLP group mice. (D) Heatmap showing the differential expression of PTX3, RBM15, YTHDF2, and SOX18 in the heart of Sham and CLP group mice. (E) ChIP-PCR/qPCR results of Sham and CLP group mice heart (n = 6). (F) Dot blot results. (G) m6A modification status of all mRNAs in the heart of Sham and CLP group mice. (H) m6A modification status of SOX18 mRNA in the heart of Sham and CLP group mice. (I) MeRIP-qPCR results of SOX18 mRNA in the heart of Sham and CLP group mice (n = 6). (J) RIP-qPCR results of SOX18 mRNA in the heart of Sham and CLP group mice (n = 6). (K) Immunoblot results for heart tissues from different groups of mice (n = 6). (L) Echocardiographic results from mice in different groups (n = 6). (M) ChIP-PCR/qPCR results of heart from CLP and CLP+AAV-SOX18 groups (n = 6). (N) MeRIP-qPCR results of SOX18 mRNA in the heart of CLP and CLP+AAV-shRBM15 groups (n = 6).

Considering that the CLP model is also a classic SIC model and more clinically relevant, we performed the CLP-induced mouse model and validated the findings previously observed in the LPS-induced SIC model. We conducted RNA-Seq experiments on heart tissues from the Sham and CLP groups (**Figure 7A**). GSEA results showed significant enrichment of the pyroptosis pathway in the CLP group (**Figure 7B**), and heatmap analysis revealed elevated levels of core pyroptosis-related molecules, including NLRP3 and GSDMD (**Figure 7C**). Importantly, we observed increased expression of RBM15, YTHDF2, and PTX3, alongside decreased SOX18 expression in the heart tissues of CLP mice (**Figure 7D**), consistent with our observations in LPS-induced models.

To investigate the molecular mechanisms, we performed ChIP-qPCR experiments, which demonstrated suppressed binding of SOX18 to the PTX3 promoter region in the CLP model (**Figure 7E**). To determine whether SOX18 expression in the CLP model is influenced by m6A modifications, we performed Dot blot analyses, confirming an overall increase in m6A modification levels in the CLP group (**Figure 7F**). To further explore the mechanism of SOX18 m6A modification, we conducted MeRIP-seq, which showed increased m6A modification in the 5'UTR, CDS, and 3'UTR regions of total RNA in CLP mice (**Figure 7G**). Visualization of m6A modifications revealed significantly elevated m6A levels in the CDS region of SOX18 in the CLP group compared to the Sham group. The enriched sites aligned with those identified in the LPS-induced model (**Figure 7H**). MeRIP-qPCR further validated these findings (**Figure 7I**), and RIP-qPCR confirmed increased binding of RBM15 to SOX18 mRNA in the CLP group (**Figure 7J**).

Using PTX3-cKO mice and tools such as AAV-cTnT-SOX18/shRBM15/shYTHDF2, we regulated key targets in the CLP model. Results showed that PTX3 knockout effectively suppressed pyroptosis in myocardial tissues. SOX18 overexpression reduced PTX3 expression and inhibited pyroptosis. Knocking down RBM15 or YTHDF2 restored SOX18 expression and reduced pyroptosis (**Figure 7K**). Echocardiography confirmed that SOX18 overexpression or interference with PTX3/RBM15/YTHDF2 significantly improved cardiac function in CLP mice (**Figure 7L**). SOX18 overexpression also restored its binding to the PTX3 promoter (**Figure 7M**). Furthermore, RBM15 knockdown reduced m6A modification levels of SOX18 mRNA in the myocardium of CLP mice (**Figure 7N**).

In summary, we have validated our previous findings in the CLP model: elevated RBM15 and YTHDF2 in CLP mice promote m6A modification of SOX18, leading to its downregulation. Low SOX18 levels fail to transcriptionally repress PTX3, resulting in myocardial pyroptosis and impaired cardiac function.

## Discussion

In this study, we have made three key findings. First, we identified PTX3 as a critical component of the NLRP3 inflammasome. PTX3 is significantly upregulated during the progression of SIC and triggers pyroptotic responses. Inhibition of PTX3 effectively mitigates cardiomyocyte pyroptosis in SIC. Second, we discovered that the downregulation of SOX18 in SIC is a major factor contributing to the increased expression of PTX3. Under physiological conditions, SOX18 binds to the promoter region of PTX3 and suppresses its transcription. However, during SIC, the reduced expression of SOX18 results in the failure to effectively inhibit PTX3 elevation. Third, we demonstrated that the downregulation of SOX18 in SIC is mediated by RBM15/YTHDF2. RBM15 catalyzes the m6A modification of SOX18 mRNA, and YTHDF2 recognizes this m6A modification, reducing the stability of SOX18 mRNA, which leads to decreased SOX18 expression.

PTX3 is an acute-phase protein and a member of the pentraxin family, playing a crucial role in immune regulation. Synthesized and stored in various cell types, current evidence suggests that PTX3 serves as a key mediator at the intersection of inflammation, immunity, tissue repair, and tumorigenesis. In cardiovascular diseases, PTX3 contributes to disease progression through mechanisms such as exacerbating endothelial dysfunction, influencing angiogenesis, and regulating inflammation and oxidative stress^16^, ^17^. Furthermore, PTX3 levels are positively correlated with the risk of adverse outcomes in patients with coronary artery disease^18^, indicating its potential as a biomarker for cardiovascular conditions. Literature reports indicate that PTX3 is a significant factor contributing to cardiac dysfunction in various heart diseases. For instance, PTX3 may exacerbate trastuzumab-induced cardiac complications by inducing cardiomyocyte contractile dysfunction^19^. Additionally, PTX3 has been reported to cause cardiac electrophysiological disturbances, mitochondrial dysfunction, and cardiomyocyte apoptosis^20^. The knockout of PTX3 has been shown to promote recovery after myocardial infarction by inhibiting fibrosis^21^. However, other studies suggest that PTX3 may exhibit cardioprotective effects under certain conditions, such as providing cardioprotection in hypertensive rats^22^ and alleviating hypoxia/reoxygenation (H/R) injury in H9C2 cells^23^. Given these conflicting findings, it is imperative to investigate the role and mechanisms of PTX3 in SIC with greater caution. Building on these findings and our transcriptomic analysis, we investigated the role of PTX3 in the mechanism of cardiomyocyte pyroptosis. Our research revealed that PTX3 is involved in the assembly of the NLRP3 inflammasome, thereby inducing pyroptosis and inflammation, which contributes to the impairment of cardiac function in SIC. However, the precise regulatory mechanisms of PTX3 in SIC require further investigation.

SOX18 is a transcription factor that plays a critical regulatory role in the development of the heart and vascular system^24^, ^25^. Mutations or abnormal expression of the SOX18 gene are associated with various cardiac diseases, including congenital heart defects^26^. Additionally, SOX18 has been shown to have a protective role in sepsis, with its levels potentially serving as a prognostic marker for sepsis outcomes^14^. Our sequencing results confirmed that SOX18 is one of the most significantly downregulated transcription factors in cardiomyocytes during SIC. Subsequent experiments demonstrated that SOX18 binds to the promoter region of PTX3, inhibiting its transcription, and thereby suppressing inflammation and pyroptosis. However, during SIC, the abnormal downregulation of SOX18 diminishes this protective effect. Therefore, exploring the upstream mechanisms that regulate SOX18 is of critical importance.

M6A is an internal modification found on eukaryotic mRNA, representing a form of RNA methylation. This modification occurs at the nitrogen atom in the 6th position of the adenosine (A) base in RNA molecules, forming M6A by the addition of a methyl group to the adenosine. M6A is one of the most abundant internal modifications on eukaryotic mRNA and plays a crucial role in various processes, including RNA stability, splicing, nuclear export, translation, and degradation^27^. M6A modification has been demonstrated to play significant roles in various cardiovascular diseases, including heart failure^28^, atherosclerosis^29^, and diabetic cardiomyopathy^30^. In our study, we observed that both the overall M6A modification levels and the M6A modification levels on SOX18 mRNA were elevated in the myocardium of SIC. This modification is mediated by the M6A writer RBM15 and recognized by the M6A reader YTHDF2, ultimately leading to decreased stability of SOX18 mRNA and reduced SOX18 protein levels.

It is noteworthy that changes in SOX18 mRNA levels in SIC may also be regulated by other transcription factors (e.g., NF-kappaB)^31^ and epigenetic modifications such as DNA methylation^32^. Similarly, as the m6A modification site of SOX18 is located within its CDS region, it may influence SOX18 protein expression through various mechanisms, including promoting translation initiation^33^, causing translation stalling^34^, or recruiting translation factors^35^. We have only demonstrated that the m6A modification of SOX18 is an effective therapeutic target in SIC treatment; other underlying mechanisms still require further in-depth investigation.

Regarding the mouse SIC model, there are both LPS-induced^4^ and CLP-induced^36^ SIC models. In this study, we have validated the key conclusions in both of these models. Using strain analysis, including GLS (Global Longitudinal Strain), to assess cardiac function may be a superior indicator^37^. In the extraction and study of primary cardiomyocytes, there are different approaches, including extraction from adult mouse hearts and neonatal mouse hearts. The advantage of using adult mice for extraction is the better maturity and stability of the cardiomyocytes, but the extraction process is more challenging. In contrast, neonatal mouse extraction offers the advantages of higher cell viability, easier extraction, and lower risk of contamination. However, it requires the use of a larger number of mice and there are certain physiological differences between neonatal and adult mouse cardiomyocytes. In this study, we employed neonatal mice for cardiomyocyte extraction, and in subsequent research, we plan to explore different extraction methods to assess their differences, and we aim to introduce the Ionoptix system to conduct a more in-depth study of the contraction function and calcium dynamics changes in SIC cardiomyocytes^38^.

## Conclusion

In summary, we have delineated the RBM15/YTHDF2-SOX18-PTX3 axis in SIC. The abnormally elevated levels of RBM15 and YTHDF2 in SIC cardiomyocytes mediate the m6A modification of SOX18, leading to the downregulation of SOX18 expression. The reduced levels of SOX18 fail to effectively repress PTX3 transcription, resulting in elevated PTX3 levels that contribute to inflammasome formation and pyroptosis, further exacerbating cardiac dysfunction.

## Methods

### Animals

This study was approved by the Institutional Animal Care and Use Committee of the Air Force Medical University. Eight-week-old SPF-grade C57BL/6J mice were purchased from the Experimental Animal Center of the Air Force Medical University. The mice were housed in a controlled environment with a 12-h light/dark cycle and provided with adequate food and water. The LPS-induced SIC model was established by intraperitoneal injection of LPS (6.67 mg/kg, 72 h, or 10mg/kg, 24h). After the corresponding assessments, the mice were euthanized. The CLP-induced SIC model was established following the method described in the literature.^39^ The corresponding assessments were performed at 24 h, and the mice were then euthanized.

### Isolation and culture of primary mouse cardiomyocytes

Neonatal (1-3 days old) SPF-grade C57BL/6J mice were obtained from the Experimental Animal Center of the Air Force Medical University. The primary cardiomyocyte extraction method follows the procedure described in the reference^40^. The *in vitro* SIC model was simulated in primary cardiomyocytes using LPS at a final concentration of 5 μg/ml, and the cells were cultured for 48 h.

### siRNA/plasmid/adeno-associated virus transfection

For cell transfection, siRNA or plasmids were transfected using Lipofectamine™ 2000 (11668019, Thermo Fisher Scientific, USA) or jetPRIME transfection reagent (101000015, Polyplus, France) according to the manufacturer's instructions. For in vivo studies, adeno-associated virus (AAV) was administered via tail vein injection with a viral titer of 1.8 × 10¹² vg/ml.

### RNA-sequencing

Total RNA was extracted using Trizol reagent (thermofisher, 15596018), and its quantity and purity were assessed with a Bioanalyzer. High-quality RNA (RIN > 7.0) was used for library construction. mRNA was purified from total RNA, fragmented, and reverse-transcribed into cDNA. U-labeled second-stranded DNA was synthesized, followed by adapter ligation and size selection. The DNA was then amplified by PCR and the cDNA library with an average insert size of 300±50 bp was sequenced using 2×150bp paired-end sequencing on an Illumina Novaseq™ 6000.

### Echocardiography

Mice were anesthetized and placed in a supine position. The dimensions of the left ventricle, including end-diastolic and end-systolic diameters, were measured from the parasternal short-axis view. Left ventricular ejection fraction (LVEF) and fractional shortening (FS) were calculated based on these measurements to assess the cardiac systolic function. To evaluate diastolic function, The E and A wave data were obtained through pulsed-wave Doppler, while the E' and A' wave data were obtained through tissue Doppler.

### TUNEL assay

For tissue sections: Deparaffinize the tissue sections, followed by antigen retrieval using Proteinase K. Next, treat the sections with a membrane permeabilization solution. Incubate the sections in TUNEL reaction mixture (G1502, Servicebio, China). After incubation, counterstain with DAPI and mount the sections with an anti-fluorescence quenching agent. Capture images using a fluorescence microscope (Nikon Eclipse C1, Nikon, Japan).

For cell slides: According to the manufacturer's instructions (G1502, Servicebio, China), apply the permeabilization working solution to the cell slides and incubate for 20 min at room temperature. Then, apply the TUNEL reagent to the slide, ensuring it covers the cells, and incubate at 37°C for 2 h. Finally, mount the slides with an anti-fluorescence quenching medium containing DAPI (S2110, Solarbio, China), and capture images using a fluorescence microscope (Nikon Eclipse C1, Nikon, Japan).

### qPCR assay

Total RNA was extracted using TRIzol reagent (15596018, Thermofisher, USA). The extracted RNA was then reverse transcribed into cDNA. The resulting cDNA was amplified using specific primers (Sangon, China) and 2× Universal Blue SYBR Green qPCR Master Mix (G3326, Servicebio, China). The amplification was carried out on a CFX Connect Real-Time PCR Detection System (Bio-Rad, China). The sequences of the primers (F, R; 5'-3') are as follows:

PTX3: CTGCCCGCAGGTTGTGAA, TGGTCTCACAGGATGCACG.

NLRP3: CAAGGCTGCTATCTGGAGGAA, TGCAACGGACACTCGTCATC.

SOX18: TGAGCAAGATGCTGGGCAAAG, GCGAGGCCGGTACTTGTAGT.

HIC1: TCCCCTAACCGGGGCAA, CCAGCACACTCTCCCGATTTA.

ATF3: GTCACCAAGTCTGAGGCGG, GTTTCGACACTTGGCAGCAG.

FOXF1: CAAGCAACAGCCTCTGTCCC, TACCGAGGGATGCCTTGCAG.

METTL3: CTTGCCATCTCTACGCCAGA, TCTTGGAGGAGACCTCGCTT.

METTL14: TATGCTTGCGAAAGTGGGGT, CCATCAGGCAATGCTCCTTTG.

WTAP: AAAGCAGCAACAGCAGGAGT, GGTACTGGATTTGAGTGGTGC.

RBM15: TCATGCCTTCCCACCTTGTG, GTTCACCAGTTTTGCACGGA.

ALKBH5: TGACTGTGCTCAGTGGGTATG, CCTGAGAATGATGACCGCCC.

FTO: CTTCACCAGGGAGACTGCTAT, GGTGCCTGTTGAGCACTCTG.

YTHDF1: AGGTGGTGCGTAAGGAAAGAC, GGAGACAGCACCAAGCATACA.

YTHDF2: CACAGGCAAGGCCGAATAAT, ACCAAGCAGCTTCACCCAAA.

YTHDC1: GCCGGGAGGAGAAAGATGG, ATACAATTCATCATCCTGTTCTGGT.

IGF2BP2: CTGGCCGTTAACCAACAAGC, GCACAGACAGTCCAGTCGAA.

B-ACTIN: CACTGTCGAGTCGCGTCC, CGCAGCGATATCGTCATCCA.

### Western blotting

Proteins from tissues or cells were extracted using RIPA lysis buffer (P0013C, Beyotime, China) and quantified. The protein samples were mixed with SDS-PAGE sample buffer (P0015L, Beyotime, China) and denatured at 100°C. Following electrophoresis, the proteins were transferred to a membrane, blocked, incubated with primary antibodies, and subsequently with secondary antibodies. Detection was performed using an appropriate chemiluminescence reagent. The details of the antibodies used are as follows: PTX3: 13797-1-AP, Proteintech, China; NLRP3, 68102-1-Ig, Proteintech, China; Caspase1: 22915-1-AP, Proteintech, China; GSDMD: ab209845, Abacm, UK, Beta Actin: 66009-1-Ig, Proteintech, China; SOX18: ab66145, Abacm, UK; METTL3: 15073-1-AP, Proteintech, China; METTL14: 26158-1-AP, Proteintech, China; WTAP: 60188-1-Ig, Proteintech, China; RBM15: 10587-1-AP, Proteintech, China; YTHDF1: 17479-1-AP, Proteintech, China; YTHDF2: 24744-1-AP,Proteintech, China; YTHDC1: 14392-1-AP, Proteintech, China; IGF2BP2: ab124930, Abacm, UK.

### Immunohistochemistry

Paraffin-embedded tissue sections were deparaffinized and subjected to antigen retrieval. Endogenous peroxidase activity was blocked, and sections were subsequently incubated with serum blocking solution (GC305010, Servicebio, China). After overnight incubation with primary antibodies at 4°C, secondary antibodies were applied and incubated at room temperature. DAB staining was performed to visualize the antigen, followed by nuclear counterstaining (G1212, Servicebio, China). The sections were then dehydrated, cleared, and mounted with a mounting medium. Images were captured using a microscope (E100, Nikon, Japan).

### Scanning electron microscopy

Cell coverslips were rinsed with PBS and then fixed with glutaraldehyde (G1102, Servicebio, China). After fixation, samples were treated with 0.1M phosphate buffer (pH 7.4) and subjected to a graded dehydration series in ethanol (30%-100%). Following dehydration, samples were processed in a critical point dryer (K850, Quorum, UK) and then gold-coated (MC1000, Hitachi, Japan). Finally, images were captured using a scanning electron microscope (SU8100, Hitachi, Japan).

### Flow cytometry

Cells were digested and centrifuged, then resuspended in PBS. Following the instructions of the Green Fluorescent FAM-FLICA® Caspase-1 (YVAD) Assay Kit (Kit 97, Immunochemistry, USA), cells were double-stained with Active-caspase-1 and PI. Flow cytometric analysis was performed using a flow cytometer (DxFlex, Beckman Coulter, USA).

### Confocal microscopy

Cells were plated in confocal dish plates and fixed with paraformaldehyde. After blocking and permeabilization, cells were incubated with primary antibodies followed by fluorescence-conjugated secondary antibodies (SA00003/SA00009, Proteintech, China). After mounting with an antifade reagent containing DAPI (S2110, Solarbio, China), images were acquired using a confocal microscope (FV3000, Olympus, Japan).

### Co-immunoprecipitation (CoIP) assay

Cell lysates were prepared according to the instructions of the Co-immunoprecipitation Kit (abs955, Absin, China). After pre-clearing, specific primary antibodies against the target protein were added, with IgG antibodies used as controls. Following washing of the immune complexes, the precipitated protein samples were analyzed using the previously described Western blotting method.

### Chromatin immunoprecipitation (ChIP) assay

ChIP analysis was performed using the SimpleChIP Plus Sonication Chromatin IP Kit (#56383, Cell Signaling Technology, USA). Initially, DNA and proteins were cross-linked and sonicated into small fragments. DNA fragments were then captured using either specific antibodies against the target protein or IgG as a control. The captured DNA was subsequently purified and subjected to PCR and qPCR analyses. PCR products were analyzed by agarose gel electrophoresis, and fold enrichment was calculated from the CT values obtained from qPCR.

### Dot blot assay

Total RNA was extracted using TRIzol (15596018, Thermofisher, USA) and diluted with nuclease-free water to concentrations of 200 ng/μL and 100 ng/μL. The RNA was heated in a water bath at 70°C, then dot-blotted onto a nylon membrane under a nuclease-free environment. After UV crosslinking, the membrane was blocked with 5% BSA and incubated with an anti-m6A antibody (1D5E10, Proteintech, China). Following incubation with a secondary antibody, the membrane was developed using ECL. Methylene blue staining (0.02%) was performed for loading control visualization.

### RIP/MeRIP assay

RIP/MeRIP assays were performed using the RIP/MeRIP Kit (Bes5101/Bes5203, Bersinbio, China). After RNA extraction and quality control, the RNA was fragmented. Immunoprecipitation was then conducted using antibodies against the target protein, m6A, or IgG as a control.

### RNA stability assay

Cells were divided into 0-6h groups. Actinomycin D (HY-17559, MCE, China) was added to the culture medium at a final concentration of 10 µg/ml to inhibit DNA transcription into RNA. RNA was extracted at each designated time point, and RNA stability was assessed by comparing the levels of RNA extracted at each time point with those from the 0h group using qPCR.

### Dual-luciferase reporter assay

Cells were transfected with luciferase reporter plasmids containing either wild-type or mutant SOX18 mRNA sequences, along with either an empty vector or oe-RBM15 plasmid. The ratio of firefly luciferase (primary reporter) to Renilla luciferase (internal control) was used to assess the stability of SOX18 mRNA.

### Serum collection and analysis

Following anesthesia, blood was collected from the retro-orbital plexus of the mice. The collected blood was transferred to centrifuge tubes and subjected to centrifugation at low temperature (4°C, 3000 rpm for 10 min). The upper serum layer was carefully collected and analyzed using a biochemical analyzer.

### Statistical analysis

Statistical analysis was performed using GraphPad Prism 9 software. For continuous variables that followed a normal distribution, Student's t-test was used; for variables that did not follow a normal distribution, the Mann-Whitney U test was employed. Analysis of variance (ANOVA) was used to compare two or more variables across multiple groups. Pearson chi-square or Fisher's exact test was applied for categorical variable comparisons. Results are presented as mean ± SEM. A p-value less than 0.05 was considered statistically significant.

## Supplementary Material

Supplementary figures and tables.

## Abbreviations

SIC: sepsis-induced cardiomyopathy
PTX3: Pentaxin 3
SOX: SRY-related HMG-box
RBM15: RNA binding motif protein 15
YTHDF2: YTH N6-methyladenosine RNA binding protein 2
LVEF: left ventricular ejection fraction
FS: fractional shortening
CO: cardiac output
CLP: cecal ligation and puncture
AAV: Adeno-associated virus
