# Supplementary material for: The m6A modification of SOX18 leads to increased PTX3 and cardiomyocyte pyroptosis in sepsis-induced cardiomyopathy
Source: Theranostics. 2025 Feb 24;15(8):3532–50. doi: 10.7150/thno.103809 (PMC11905121; doi:10.7150/thno.103809)
Supplement: Supplementary file 1 — Supplementary figures and tables. [file thnov15p3532s1.pdf]

Supplemental figures

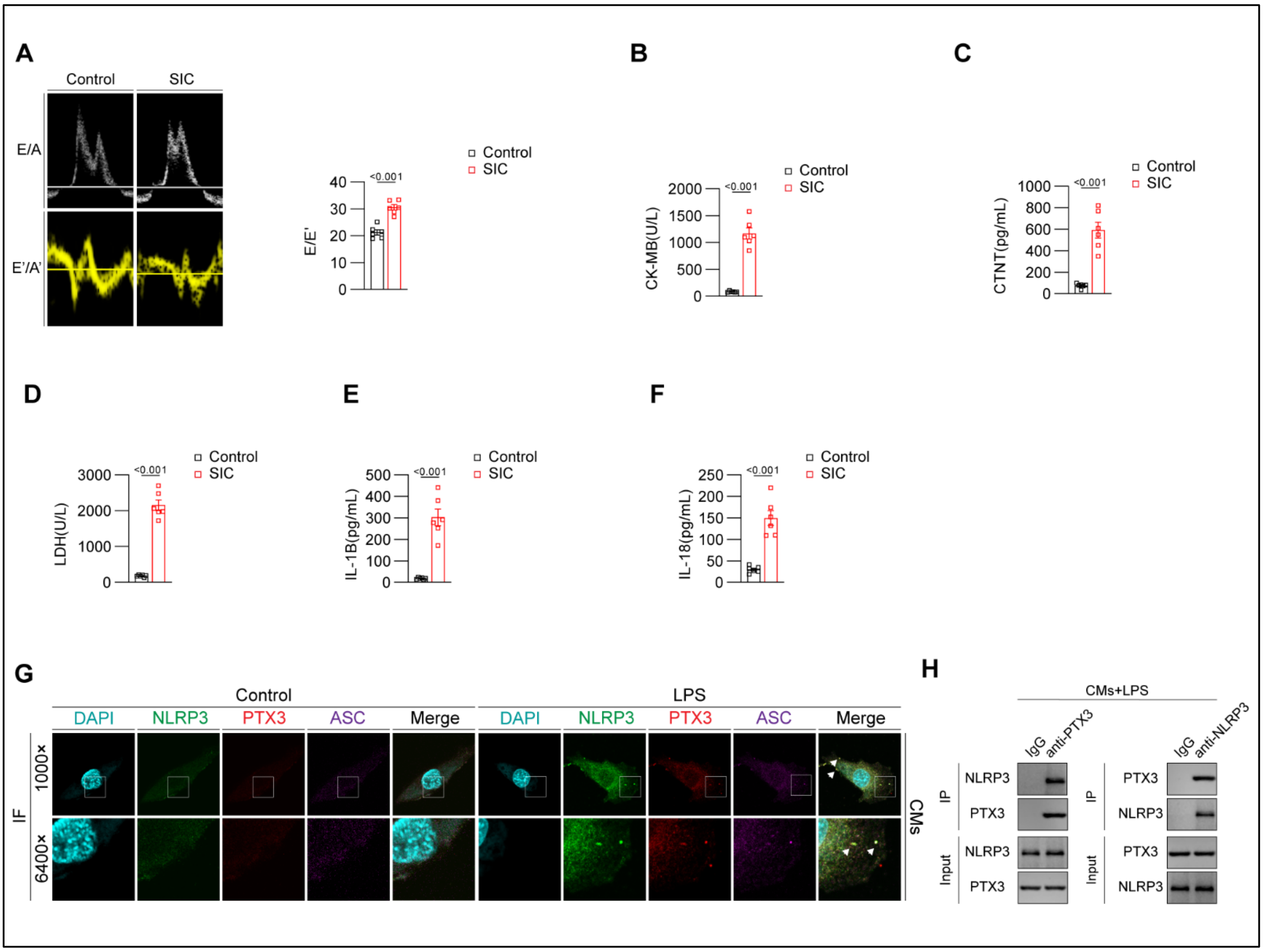

Figure S1

(A) Measurement of diastolic function in mice (n = 6). (B) Serum CK-MB levels in mice (n = 6). (C) Serum CTNT levels in mice (n = 6). (D) Serum LDH levels in mice (n = 6). (E) Serum IL-1β levels in mice (n = 6). (F) Serum IL-18 levels in mice (n = 6). (G) Confocal microscopy observation of NLRP3, PTX3 and ASC localization in primary cardiomyocytes. The white arrow points to the co-localization of the three proteins. (H) Co-IP assay showing the interaction between PTX3 and NLRP3 in primary cardiomyocytes.

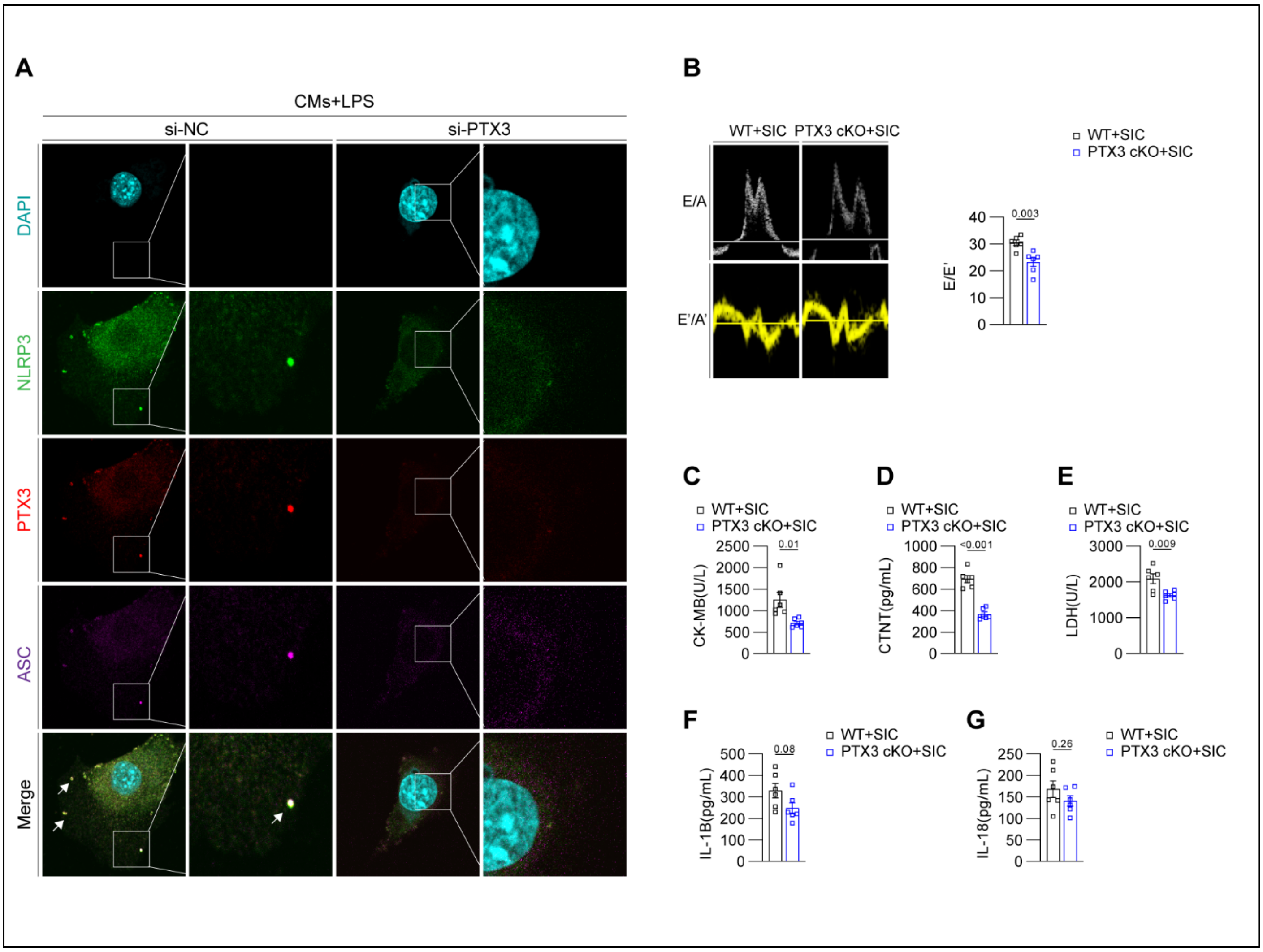

Figure S2

(A) Confocal microscopy observation of NLRP3, PTX3 and ASC localization in primary cardiomyocytes. The white arrow points to the co-localization of the three proteins. (B) Measurement of diastolic function in mice (n = 6). (C) Serum CK-MB levels in mice (n = 6). (D) Serum CTNT levels in mice (n = 6). (E) Serum LDH levels in mice (n = 6). (F) Serum IL-1β levels in mice (n = 6). (G) Serum IL-18 levels in mice (n = 6).

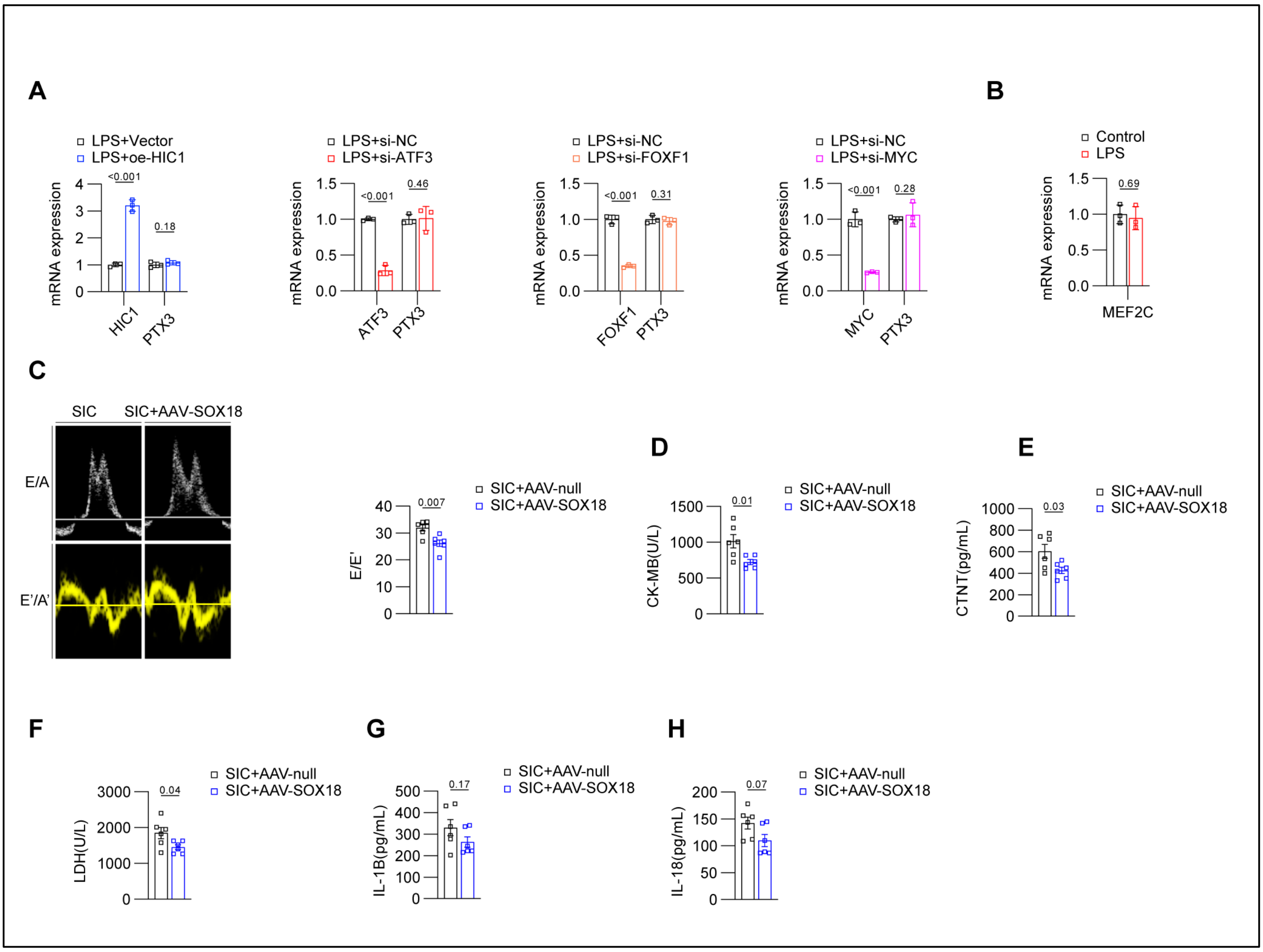

Figure S3

(A) qPCR experiment to screen potential transcription factors that may regulate PTX3. (B) qPCR analysis of the expression of MEF2C. (C) Measurement of diastolic function in mice (n = 6). (D) Serum CK-MB levels in mice (n = 6). (E) Serum CTNT levels in mice (n = 6). (F) Serum LDH levels in mice (n = 6). (G) Serum IL-1β levels in mice (n = 6). (H) Serum IL-18 levels in mice (n = 6).

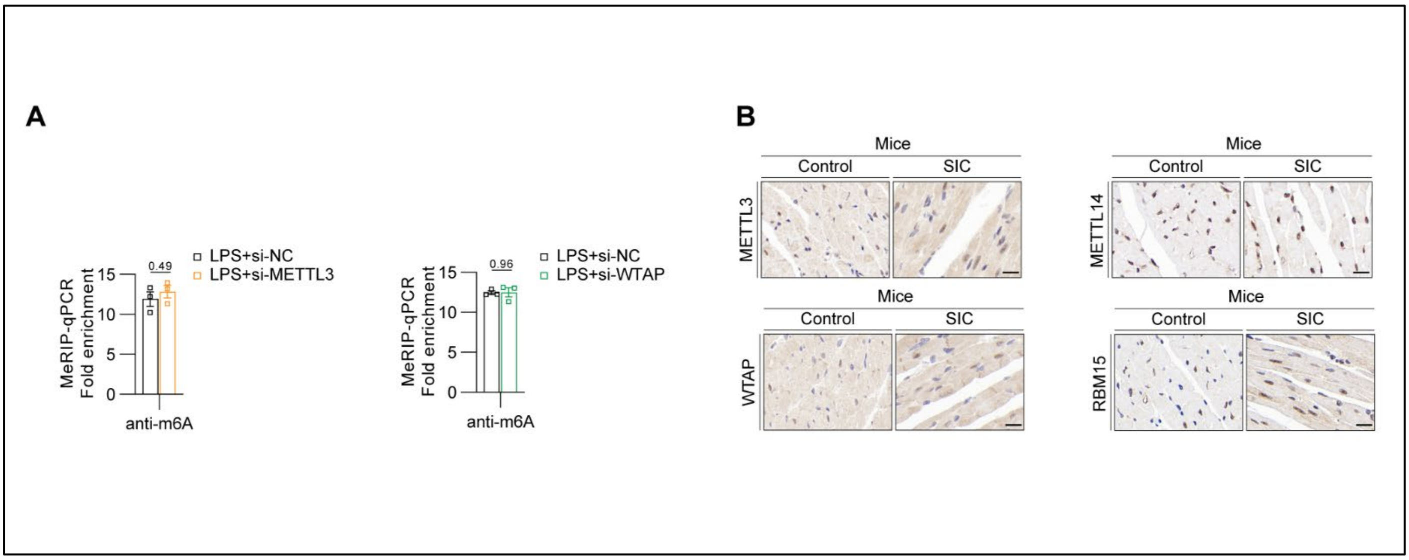

Figure S4

(A) MeRIP-qPCR experiment to observe the m6A modification status of SOX18 after interference with METTL3 or WTAP (n = 3). (B)Immunohistochemical analysis of METTL3, METTL14, WTAP, and RBM15 protein expression in mouse cardiac tissue. Scale bar: 20μm.

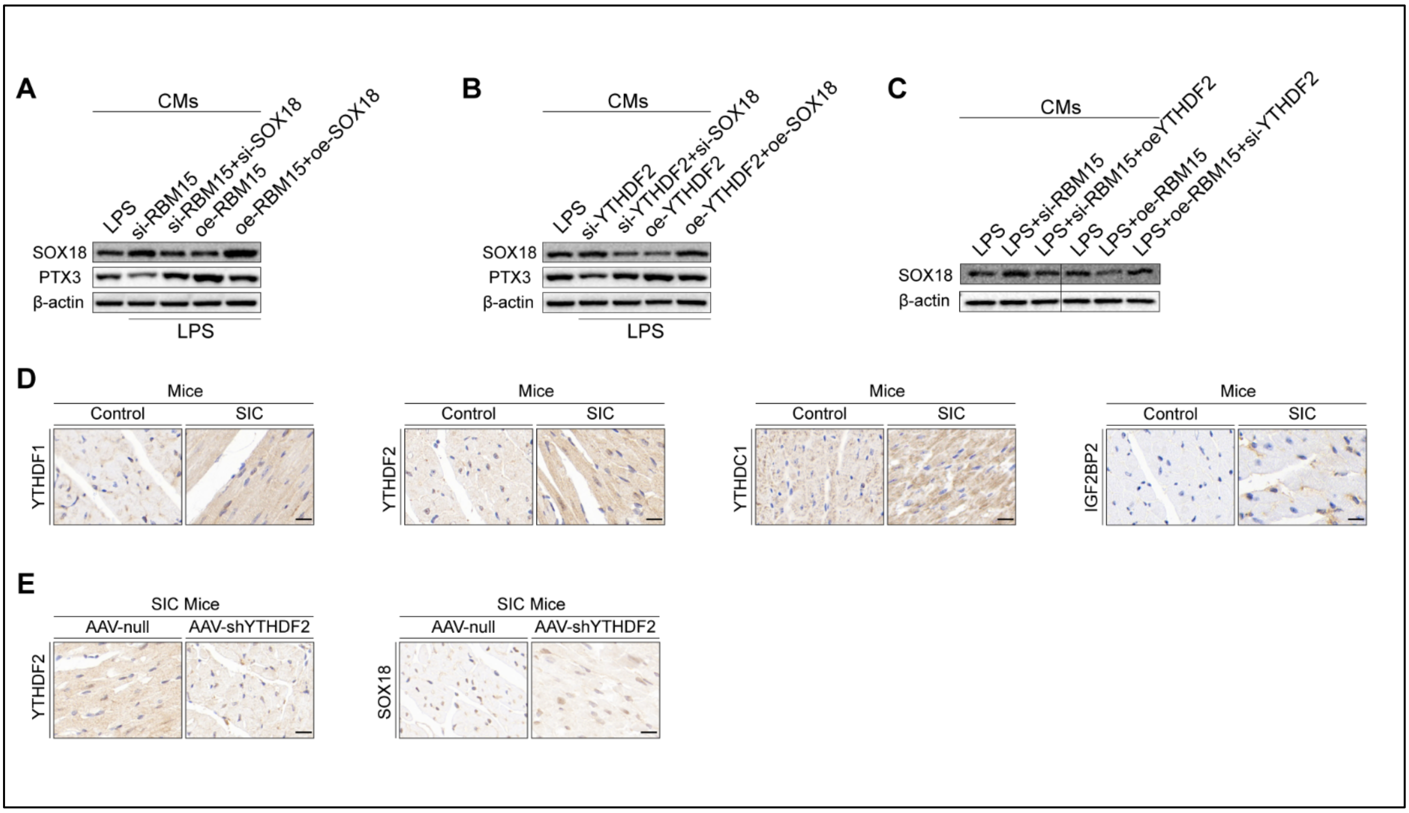

Figure S5

(A) Western blot detection of PTX3 changes after intervening with SOX18/RBM15 in LPS-stimulated cardiomyocytes. (B) Western blot detection of PTX3 changes after intervening with SOX18/YTHDF2 in LPS-stimulated cardiomyocytes. (C) Western blot detection of SOX18 changes after intervening with RBM15/YTHDF2 in LPS-stimulated cardiomyocytes. (D) Immunohistochemical analysis of YTHDF1, YTHDF2, YTHDC1, and IGF2BP2 protein expression in mouse cardiac tissue. Scale bar: 20μm. (E) Immunohistochemical analysis of YTHDF2 and SOX18 protein expression in mouse cardiac tissue. Scale bar: 20μm.

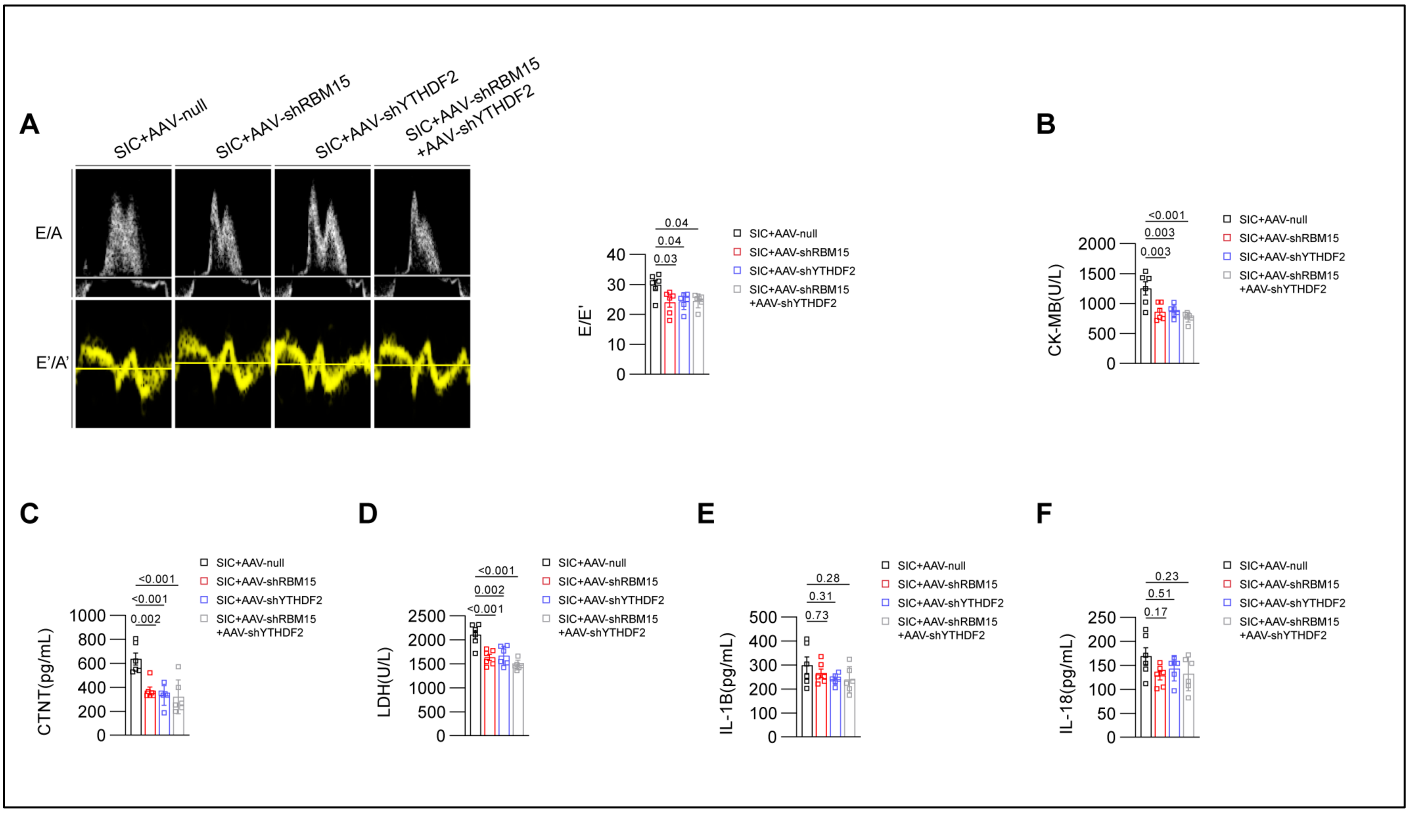

Figure S6

(A) Measurement of diastolic function in mice (n = 6). (B) Serum CK-MB levels in mice (n = 6). (C) Serum CTNT levels in mice (n = 6). (D) Serum LDH levels in mice (n = 6). (E) Serum IL-1β levels in mice (n = 6). (F) Serum IL-18 levels in mice (n = 6).
